# Occupational violence toward mental health professionals during the COVID-19 pandemic: an international study

**DOI:** 10.3389/fpsyt.2024.1527182

**Published:** 2025-01-20

**Authors:** Rebeca Robles, Cary S. Kogan, María Elena Medina-Mora, José Ángel García-Pacheco, Tahilia J. Rebello, Brigitte Khoury, Maya Kulygina, Geoffrey M. Reed

**Affiliations:** ^1^ Center for Global Mental Health Research, Ramón de la Fuente Muñiz National Institute of Psychiatry/UNAM, Mexico City, Mexico; ^2^ School of Psychology, University of Ottawa, Ottawa, ON, Canada; ^3^ Faculty of Psychology, National Autonomous University of Mexico, Mexico City, Mexico; ^4^ Departamento de Administración Pública, Centro de Investigación y Docencia Económica, Mexico City, Mexico; ^5^ Department of Psychiatry, Columbia University Vagelos College of Physicians and Surgeons, New York, NY, United States; ^6^ Department of Psychiatry, American University of Beirut Medical Center, Beirut, Lebanon; ^7^ Training and Research Centre, Mental-health Clinic No.1 named after N.A. Alekseev, Moscow, Russia

**Keywords:** occupational violence, distress, mental health professionals, risk factors, COVID-19

## Abstract

**Introduction:**

According to pre-COVID-19 pandemic studies, occupational violence (OV) toward mental healthcare professionals (MHCPs) is a common phenomenon with important consequences for their own mental health. This study sought to assess the prevalence of different types and sources of OV toward MHCPs during the COVID-19 pandemic, analyze the risk for OV conferred by relevant factors, and compare the emotional distress reported by MHCPs with and without OV.

**Methods:**

The study is an international cross-sectional Internet-based study completed by 3,325 MHCPs having provided direct clinical services during the COVID-19 pandemic.

**Results:**

13.11% experienced OV. The most frequent type/source of OV was psychological violence inside the workplace (59.6% of those who reported OV). Risk factors for any type/source of OV being younger, working in emergency services, treating COVID-19 patients, and living in a lower to upper middle-income country. Emotional distress was higher in those who had experienced OV. Risk factors for emotional distress among those reporting OV included being younger and having experienced physical violence outside the workplace.

**Discussion:**

Approximately one in ten MHCP experienced OV during the COVID-19 pandemic. This figure is consistent with the range of OV against MHCPs reported prior to the pandemic and indicates that efforts are needed to prevent and manage OV and its negative emotional consequences among MHCP, particularly in aforementioned high-risk groups during health emergencies, and addressing both proximal and distal environmental factors related to OV toward MHCPs.

## 1 Introduction

Occupational violence (OV) against healthcare workers is an increasing global concern, with evidence suggesting that its prevalence increased significantly during the COVID-19 pandemic (1–4). According to Wynne et al. (5), OV includes “incidents where staff are abused, threatened or assaulted in circumstances related to their work, including commuting to and from work, involving an explicit or implicit challenge to their safety, well-being or health.” It encompasses both psychological and physical violence. Regarding the source of violence, OV has been studied more extensively inside than outside the workplace. However, during the COVID-19 pandemic, numerous reports of OV targeting healthcare workers outside the workplace prompted researchers to understand, prevent, and address this phenomenon, especially in the context of infectious disease outbreaks (3, 6–9).

The Theoretical Framework for OV toward Healthcare Workers include Poyner and Wame (10) model of OV, which has been used to build a profile of high-risk situations in general health care, including emergency departments (11) and psychiatric settings (12). It posits that there are not only individual factors, but also interpersonal and situational factors that are crucial to the generation of OV, underlining the need for research focusing on interaction behaviors and specific contextual situations in violent incidents rather than the individual characteristics of those interacting in these events, such as gender. The Social-Ecological Model, which also includes individual, relationship, and societal factors, has been considered helpful for identifying and implementing effective OV prevention strategies (13). Regarding the role of environmental factors, the Negative Affect Escape Model proposes that unpleasant environmental stimuli increasing in intensity can often lead to aggression (14), while the Excitation-Transfer Theory states that one stimulus can build on another, triggering situations that can lead to aggressive behavior (15). These models have also been used to understand OV toward healthcare workers (16). Considering this theoretical framework, in addition to the well-known sociodemographic and professional variables related to OV, contextual factors in stressful events (such as a worldwide health emergency like COVID-19) should be addressed to identify vulnerable groups of healthcare workers requiring primary to tertiary preventive measures (see for example: 17).

Prior to the pandemic, OV toward healthcare workers was reported to be relatively commonplace. According to a 2019 meta-analysis, 12-month prevalence of any type of OV committed by patients or visitors against healthcare workers was 61.9% (95% CI= 56.1-67.6), with psychological violence being the most common form of violence (42.5%), particularly verbal abuse (18). Prevalences varied substantially across countries, occupation, and practice settings, with a higher proportion of OV being experienced by healthcare workers in Asian and North American countries, among nurses and physicians, and in psychiatry and emergency services (18). OV has also been associated with significant negative personal and professional effects. For example, according to a study by Rosenthal et al. (19), sixty percent of healthcare workers reporting any form of OV had experienced at least one posttraumatic symptom, while thirty percent had entertained thoughts about leaving their jobs or careers because of violence. According to Lanctôt and Guay’s (20) systematic literature review of OV toward healthcare workers, emotional distress is one of the most frequent and important effects of OV. Studies comparing the mental health consequences of OV according to the type of violence experienced reveal that the percentage of healthcare workers reporting emotional distress is higher if the OV consisted of physical rather than psychological violence (21). However, more research on both types of violence is required to demonstrate its prevalence and impact, and to inform the development and implementation of programs to meet the needs of the health workforce (19).

Data collected during the COVID-19 pandemic has revealed wide variability in estimates for prevalence of OV, ranging from 18.5 to 84.5%. According to Jacobi and Ide (22), countries with stronger institutions were relatively safer for health workers. Being male, younger, having less job experience, and being in direct contact with COVID-19 patients were key risk factors for OV, which was mostly perpetrated by caregivers and COVID-19 patients’ family members (23). Moreover, there is evidence that during the COVID-19 pandemic, OV has triggered or intensified health care workers’ personal and professional distress, including anxiety, depression, stress-related symptoms, and burnout (24, 25), all of which are likely to exacerbate current recruitment and retention difficulties and increase workforce shortages (26).

OV and its negative consequences in Mental Health Care Professionals (MHCPs) are particularly worrisome. MHCPs experience a higher prevalence of violent events than other healthcare workers (27–29). In turn, increases in prevalence can further diminish human resources for dealing with potentially aggressive patients and their family members (30). However, there is a dearth of studies examining the psychological consequences of OV in MHCPs (31). According to a systematic review of sixteen pre-pandemic articles (32), prevalence of OV toward psychiatric nurses ranged from 11.4 to 97.6%, with those who experienced OV experiencing poorer mental health and more negative work-related outcomes. Another study found that clinical symptoms of post-traumatic stress were reported by several victims of OV, while a significant proportion (45%) had taken time off work after experiencing a violent incident (33).

Given all the above, the main objective of the present study was to assess the prevalence of different types (physical or psychological) and settings (inside or outside the workplace) of OV experienced by an international sample of MHCPs during the COVID-19 pandemic. Additional objectives included identifying significant demographic, professional and violence- or COVID-19-related factors that increase the risk of experiencing OV and related emotional distress.

## 2 Materials and methods

The current study is part of a broader internet-based survey focusing on the longitudinal impact of the COVID-19 pandemic on MHCPs’s practice and well-being. (34, 35), which was approved by the Institutional Review Board at Columbia University/New York State Psychiatric Institute and the University of Ottawa (Registration number: H-06-20-5973; principal investigators: Drs. Cary S. Kogan and Geoffrey Reed).

### 2.1 Participants

To reach a large, multilingual, international sample of psychiatrists, psychologists, and other MHCPs, a non-probabilistic convenience sample of members of the Global Clinical Practice Network (GCPN) (36), which at that time of data collection comprised approximately 16,000 MHCPs from 163 countries. GCPN members were sent an email inviting them to participate in an online study on the effects of the COVID-19 pandemic on various aspects of their professional practice and personal experience. A total of 3,986 GCPN members participated in three periods of data collection: 1) wave 1 from June 4 to July 7, 2020; 2) wave 2 from November 11 to December 18, 2020; and 3) wave 3 from July 28 to September 7, 2021. Participants from all three waves were included in the present analysis. In cases where participants responded on more than one occasion, the last response was used. On this basis, half the participants used in this analysis responded during wave three (52.6%), and the remaining half were equally distributed between wave one (23.9%) and wave two (23.5%).

### 2.2 Variables and measures

The survey comprised self-reported questions on demographic and professional characteristics, including potential covariates according to previous studies, prior to (18) and during the COVID-19 pandemic (23): gender, age, country, profession, years of professional experience, type of practice setting, and whether they provided direct care for COVID-19 patients. The World Bank classification was used to determine the income levels of each country.

Violence and COVID-19-related variables included 1) the homicide rate per 100,000 population (as a general proxy for violence), registered by each participating country based on available information between 2002 and 2020 (37), and 2) the stringency index of COVID-19 measures by country as an indicator of the severity of government responses (38). The stringency index can vary from 0 to 100 (with 100 representing the strictest measures), based on nine indicators related to government policies on closure of public spaces or activities (such as schools, workplaces, public transport and public events) (39); and 3) the COVID-19 death rate per 100,000 population, calculated by country based on confirmed deaths.

OV was evaluated through the following question, answering separately for type (physical or psychological) and source (inside or outside the workplace): “During the COVID-19 pandemic, have you been the target of physical or psychological violence or maltreatment, including being stigmatized or discriminated against because of your role as a health professional”?

Emotional distress was evaluated using the Depression, Anxiety, Stress Scales-21 (DASS-21), a short form of the three DASS self-report scales designed to measure the emotional states of depression, anxiety and stress, which have demonstrated high cross-cultural internal consistency coefficients for each of the subscales, and convergent and divergent validity (40). Each scale contains seven items to be answered using a four-point severity/frequency scale (0: “Did not apply to me at all” to 3: “Applied to me very much or most of the time”) to rate the extent to which they have experienced each negative emotional state in the past week. Scores for Depression, Anxiety and stress are calculated by adding the scores for the relevant items. Because the DASS-21 is a shorter version of the original 42-item DASS, the score for each subscale must be doubled to calculate the final score (41). In the present study, we used an overall score calculated by adding the scores of the three subscales as a measure of overall emotional distress.

### 2.3 Procedures and data analyses

The survey was administered using Qualtrics™ (Provo, UT, USA) in Chinese, English, French, Japanese, Russian, and Spanish. Survey questions were developed in English and translated by experts affiliated with the GCPN International Advisory Group, which included representatives from various global regions, fluent in each of the five languages other than English.

To prevent information loss, 25 missing data points were estimated with multiple imputation using additive regression, bootstrapping, and predictive mean matching technique with five multiple imputations. Thus, estimated data were assigned for the variable homicide rate (n=1), COVID-19 deaths (n=8), stringency index (n=15) and years of experience (n=1).

We examined the frequencies and percentages of categorical variables by each type/source of OV, using chi-square tests for comparison purposes. For continuous variables, we examined the mean, standard deviation, and range, and used the student’s t-test to examine the difference in means. Finally, logistical regression models were used to examine the risk factors for experiencing OV and emotional distress. We used the Hosmer-Lemeshow test to validate the goodness of fit of the model.

We split the age sample at the mean to create an equal cut-off point between younger and older ages. Since previous analyses of our data set using age as a continuous variable had shown that young people experienced more acts of violence, we considered younger ages as an explanatory category. In relation to income groups, we combined the variable originally divided into four categories, since the initial analyses showed that low, lower-middle and upper-middle income countries experienced significant amounts of violence. We therefore combined the latter groups into a single category, used as an explanatory variable in contrast to data from participants living in high-income countries. Regarding the type of practice setting, given the high percentage of participants who reported working at both public and private facilities and in both inpatient and outpatient settings, we created dummy variables for each practice setting we evaluated that was not selected by the whole sample (such as private facilities vs. others, inpatient settings vs. others, and emergency services vs. others). WHO region and years of experience were excluded from bivariate and multivariate analyses given their co-linearity with income level and age respectively.

## 3 Results

Of the 3,925 MHCPs who completed the survey, 3,325 reported having provided direct clinical services during the COVID-19 pandemic and therefore constituted the sample for the present analysis. This sample had an average of 19.9 + 10.3 years of professional experience, and comprised MHCPs from various WHO regions, reflecting the distribution of MHCPs worldwide (42): Africa n=127, 3.8%; America-South n=475, 14.3%; America-North n=397, 11.9%; Eastern Mediterranean Region n=87, 2.6%; Europe n=1,353, 40.7%; South-East Region n=234, 7.0%; Western Pacific-Asia n=580, 17.4%; and Western Pacific-Oceania n=72, 22%.

**Table 1** shows the descriptive statistics for the remaining variables for the whole sample and by experience of each type and source of OV. 13.11% (n= 436) of the total sample experienced OV during the COVID-19 pandemic. Those reporting any type/source of OV were more likely to be men, younger, from a lower- to upper-middle income country, psychiatrists, caring for COVID-19 patients, and/or working in emergency services (as opposed to other facilities).

**Table 1:** Description and comparisons of demographic, professional and violence- or COVID-19-related variables by any and each type/source of occupational violence.

Variables	Total sample	Any type/source of violence		Psychological violence inside workplace		Psychological violence outside workplace		Physical violence inside workplace		Physical violence outside workplace	
		Yes	No	Yes	No	Yes	No	Yes	No	Yes	No
	(n=3,325)	(n=436)	(n=2,889)	(n=260)	(n=3,065)	(n=236)	(n=3,089)	(n=59)	(n=3,266)	(n=46)	(n=2,279)
Gender, *n* (%)											
Male	1,673 (100)	244 (14.6)*	1,429 (85.4)	146 (8.7)	1,527 (91.3)	135 (8.1)*	1,538 (91.9)	39 (2.3)*	1,634 (97.7)	29 (1.7)	1,644 (98.3)
Female	1,652 (100)	192 (11.6)	1,460 (88.4)	114 (6.9)	1,538 (93.1)	101 (6.1)	1,551 (93.9)	20 (1.2)	1,632 (98.8)	17 (1)	1,635 (99)
Age, *n* (%)											
24-49 years	1,627 (100)	262 (16.1)***	1,365 (83.9)	152 (9.3)**	1,475 (90.7)	157 (9.6)***	1,470 (90.4)	30 (1.8)	1,597 (98.2)	25 (1.5)	1,602 (98.5)
50-89 years	1,698 (100)	174 (10.2)	1,524 (89.8)	108 (6.4)	1,590 (93.6)	79 (4.7)	1,619 (95.3)	29 (1.7)	1,669 (98.3)	21 (1.2)	1,677 (98.8)
Income level, *n* (%)											
Low to upper-middle	1,393 (100)	221 (15.9)***	1,172 (84.1)	134 (9.6)**	1,259 (90.4)	124 (8.9)***	1,269 (91.1)	32 (2.3)	1,361 (97.7)	29 (2.1)**	1,364 (97.9)
High	1,932 (100)	215 (11.1)	1,717 (88.9)	126 (6.5)	1,806 (93.5)	112 (5.8)	1,820 (94.2)	27 (1.4)	1,905 (98.6)	17 (0.9)	1,915 (99.1)
Profession, *n* (%)											
Medicine^a^	1,705 (100)	265 (15.5)***	1,440 (84.5)	169 (9.9)***	1,536 (90.1)	131 (7.7)	1,574 (92.3)	36 (2.1)	1,669 (97.9)	25 (1.5)	1,680 (98.5)
Other^b^	1,620 (100)	171 (10.6)	1,449 (89.4)	91 (5.6)	1,529 (94.4)	105 (6.5)	1,515 (93.5)	23 (1.4)	1,597 (98.6)	21 (1.3)	1,599 (98.7)
Contact COVID-19 patients *n* (%)											
Yes	848 (100)	199 (23.5)***	649 (76.5)	120 (14.2)***	728 (85.8)	120 (14.2)***	728 (85.8)	29 (3.4)***	819 (96.6)	19 (2.2)*	829 (97.8)
No	2,477 (100)	237 (9.6)	2,240 (90.4)	140 (5.7)	2,337 (94.3)	116 (4.7)	2,361 (95.3)	30 (1.2)	2,447 (98.8)	27 (1.1)	2,450 (98.9)
Emergency services											
Yes	186 (100)	45 (24.2)***	141 (75.8)	32 (17.2)***	154 (82.8)	30 (16.1)***	156 (83.9)	10 (5.4)***	176 (94.6)	8 (4.3)***	178 (95.7)
No	3,139 (100)	391 (12.5)	2,748 (87.5)	228 (7.3)	2,911 (92.7)	206 (6.6)	2,933 (93.4)	49 (1.6)	3,090 (98.4)	38 (1.2)	3,101 (98.8)
Homicide rate^c^, mean (SD) [range]	5.7 (8.9) [.2-49.9]	5.4 (8.8) [.3-49.9]	5.7 (8.9) [.2-49.9]	5.5 (9.1) [.3-49.9]	5.7 (8.9) [.2-49.9]	5.7 (8.9) [.3-45.5]	5.7 (8.9) [.2-49.9]	4.1 (6.3) [.3-22.6]	5.7 (9.0) [.2-49.9]	8.3 (10.2)* [.3-28.7]	5.6 (8.9) [.2-49.9]
Stringency index^d^, mean (SD) [range]	59.0 (15.2) [.2-97.5]	58.2 (15.1) [15.9-92.6]	59.1 (15.2) [.2-97.5]	59.4 (14.8) [21.1-92.6]	58.9 (15.3) [.2-97.5]	56.6 (14.9)** [15.9-88.8]	59.2 (15.2) [.2-97.5]	60.4 (14.6) [30.1- 92.6]	58.9 (15.2) [.2-97.5]	56.7 (15.4) [21.1-76.7]	59.0 (15.2) [.2-97.5]
COVID19 death rate^c^, mean (SD) [range]	1.7 (2.2) [.0-19.0]	1.7 (2.4) [.0-19.0]	1.8 (2.2) [.0-11.7]	1.6 (2.4) [.0-19.0]	1.8 (2.2) [.0-19.0]	1.5 (2.2) [.0-19.0]	1.8 (2.2) [.0-19.0]	2.0 (3.2) [.0-19.0]	1.7 (2.2) [.0-19.0]	1.8 (2.0) [.0-19.0]	1.7 (2.2) [.0-19.0]

*p <.05, **p <.01, ***p <.001; ^a^94.4% were psychiatrists (n=1,609); ^b^Categories with over 1% of the sample: Psychology= 1,193 (35.9%), Counseling= 92 (2.8%), Occupational Therapy= 91 (2.7%), Social-Work= 80 (2.4%), Nursing= 63 (1.9%); ^c^Rates per 100,000 population; ^d^Value index could vary from 0 to 100 (with 100 being the strictest).

Examination of the frequency of specific types/sources of OV revealed that psychological violence inside the workplace was the most frequent type and source of OV (n= 260, 59.6% of those who reported OV), followed by psychological violence outside the workplace (n= 236, 54.1% of those who reported OV), physical violence inside the workplace (n= 59, 13.5% of those who reported OV) and physical violence outside the workplace (n= 46, 10.6% of those who reported OV). A larger proportion of MHCPs who reported physical violence outside the workplace were from countries with higher homicide rates, and most of those reporting psychological violence outside the workplace were from countries with less stringent COVID-19 containment measures (**Table 1**).

A logistical regression model including all variables as possible risk factors for any type/source of OV was found to be statistically significant for age (24-49 years; OR = 1.53, 95% CI = 1.24-1.90, p<.001); country income level (low to upper-middle income vs. high income; OR = 1.41, 95% CI = 1.10-1.82, p<.05); providing direct face-to-face services for COVID-19 patients (OR = 2.48, 95% CI = 1.98-3.10, p<.0001), and working in emergency services (as opposed to other facilities; OR = 1.50, 95% CI = 1.03-2.17, p<.05) (**Table 2**).

**Table 2:** Logistical regression model for the presence of occupational violence by demographic, professional and violence- or COVID-19-related variables (*n*=3,325).

Variables	Odds Ratios	95% CI
Intercept	.10***	.07 –.16
Age (24-49 years)	1.53***	1.24-1.90
Gender (Male)	1.09	.88-1.360.37
Profession (Medicine)	1.08	.86-1.360.47
COVID-19 patients (Yes)	2.48***	1.98-3.10
Emergency services (Yes)	1.50*	1.03-2.17
Income group (Low to upper-middle)	1.41**	1.10-1.82
Homicide rate	.98*	.97-1.00
Stringency index	.99	.99-1.00

*p <.05, **p <.01, ***p <.001; R2 Nagelkerke = .073; Hosmer-Lemeshow goodness-of-fit test p >.05.

**Table 3** presents the mean scores for emotional distress by presence or absence of any and each type/source of OV. Emotional distress was higher in those who experienced any type/source of OV, with the highest levels being found in those who experienced physical violence outside the workplace.

**Table 3:** Emotional distress (DAAS) by presence/absence of any and each type/source of occupational violence.

Variable	Total (n= 2,523)	Any type/source of violence		Psychological violence inside workplace		Psychological violence outside workplace		Physical violence inside workplace		Physical violence outside workplace	
		Yes (n= 367)	No (n=2,156)	Yes (n= 220)	No (n=2,303)	Yes (n= 201)	No (n=2,322)	Yes (n= 52)	No (n=2,471)	Yes (n= 38)	No (n=2,485)
Emotional distress, mean (SD) [range]	15.3 (16.8)	21.4 (20.2)***	14.3 (15.9)	23.1 (21.2)***	14.6 (16.2)	21 (20.0)***	15 (16.0)	24.6 (22.3)**	15.1 (16.6)	31.6 (26.2)***	15.1 (16.5)
	[0, 112]	[0, 92]	[0, 112]	[0, 92]	[0, 112]	[0, 88]	[0, 112]	[0, 76]	[0, 112]	[0, 82]	[0, 112]

**p <.01, ***p <.001.

A logistical regression model including all variables that were possible risk factors for the presence of emotional distress was found to be statistically significant for age (24 to 49 years; OR = 2.2, 95% CI = 1.28-3.89, p<.01) and physical violence outside the workplace (OR = 7.01, 95% CI = 2.28-19.57, p<.001) (**Table 4**).

**Table 4:** Logistical regression model for the presence of emotional distress outcomes by demographic, professional, violence and COVID-19 related predictors (*n*= 2,218).

Variables	Odds Ratios	95% CI
Intercept	0.01***	.00 -.02
Age (24-49 years)	2.2**	1.28 - 3.89
Gender (Male)	.99	.57 - 1.72
Profession (Medicine)	.83	.47 - 1.46
See Patients witrh Covid-19 (Yes)	1.27	.71 - 2.24
Emergency services (Yes)	1.69	.65 - 3.84
Income group (Low to upper-middle)	.61	.32 - 1.18
Homicide rate	1.02	.99 - 1.05
Stringency index	1.02	1.00 - 1.04
Psychological violence in the workplace	1.13	.45 - 2.56
Psychological violence outside the workplace	1.18	.46 - 2.69
Physical violence in the workplace	2.99	.91 - 8.73
Physical violence outside the workplace	7.01***	2.28 - 19.57

**p <.01, ***p <.001.

R2 Nagelkerke = .087; Hosmer-Lemeshow goodness-of-fit test p >.05.

## 4 Discussion

The results of the present large-scale, multilingual, international study revealed that, during the COVID-19 pandemic, MHCPs worldwide experienced various types and sources of OV, including psychological and physical violence inside and outside the workplace. Overall, at least one in ten MHCPs providing direct clinical services experienced violence during the COVID-19 pandemic because of their role as health professionals. This figure is consistent with the range of estimated OV against MHCPs reported prior to the pandemic (32), and corresponds to the minimum prevalence reported by Chirico et al. (23) in their systematic review of OV toward other healthcare workers during the COVID-19 pandemic, which affected as many as eight out of every ten healthcare workers.

### 4.1 Prevalence of OV toward MHCPs during COVID-19

Violence is a phenomenon that is expected to increase when external factors such as conditions related to the COVID-19 pandemic increase the stress levels of the population. Since the COVID-19 pandemic was a health emergency, healthcare workers and their patients and family members experienced an acute state of stress that may have exacerbated the risk for OV (23). According to several authors, the COVID-19 context could increase healthcare workers’ vulnerability to OV particularly because of a heavy workload, stressful work settings, as well as inadequate human and material healthcare resources (43–47). Additionally, healthcare workers may have experienced incidents in which they were abused, threatened or assaulted by the general public given their higher risk of being infected by, and transmitting, COVID-19 (3, 6–9).

Thus, although pre-pandemic studies suggested that OV was more common among MHCPs than other healthcare workers, given their routine contact with patients more prone to violence and aggression as well as the public stigmatization of their profession (27–29, 32), COVID-19 may have differentially increased the risk of violence among other types of healthcare workers. Consistent with this hypothesis, we found that caring for patients with a COVID-19 diagnosis doubled the risk of experiencing OV.

### 4.2 Types, sources and risk factors for OV toward MHCPs

Our results are consistent with pre- and post-pandemic studies finding that the most frequently reported type and source of OV is psychological violence occurring in the workplace (19, 23), mainly toward young physicians beginning their medical careers (23, 48). Furthermore, we found that MHCPs in low- and middle-income countries experienced more violent incidents, which may be attributable to under-resourced health systems and services (49, 50), as well as those working at emergency departments, which implicates this setting as risky for OV (18).

In line with existing theoretical frameworks (10–12, 14, 15), both individual and contextual factors interact, increasing OV toward MHCPs. This should prompt governments and other health agencies to develop targeted interventions and policies to prevent OV toward MHCPs during health emergencies by considering sociodemographic, professional and contextual vulnerability factors that increase risk of exposure to violence.

Initiatives designed to screen, assess, and manage the risk of affected patients, their families and the general population becoming violent appear to be a key goal for protecting healthcare workers during health emergencies such as COVID-19, especially the youngest ones, and those caring for affected patients and/or working at emergency departments (31). In fact, one of the main needs expressed by frontline healthcare workers during the COVID-19 pandemic was training in strategies to prevent and manage angry, hostile or aggressive patients and family members (51). However, these efforts should be complemented by the strengthening of health systems and services, particularly in low- and middle-income countries. Both proxy and distal environmental factors related to OV toward MHCPs must therefore be addressed.

### 4.3 Emotional distress as a consequence of OV toward MHCPs

We also found that MHCPs who reported experiencing OV regardless of its form or source were also more likely to report clinically and statistically significant emotional distress as compared to their counterparts who did not report this. Psychological and physical violence inside and outside the workplace was associated with harmful for MHCPs during the COVID-19 pandemic.

Importantly, according to our findings, being younger (aged 24 to 49) doubles the risk for emotional distress in those who experience OV, while physical violence outside the workplace confers a sevenfold greater risk for emotional distress in MHCPs reporting OV. This is consistent with previous studies regarding the mental health consequences of the COVID-19 pandemic among the general population and healthcare professionals in various countries that reported that being younger confers a higher risk for emotional distress (52, 53). It also accords with those comparing the mental health consequences of OV depending on the type of violence experienced (21), adding evidence of the impact of physical violence when outside work during health care emergencies, which seemed to increase during the COVID-19 pandemic (3, 6–9).

However, unlike other healthcare workers, those working in mental health may engage in fewer help-seeking behaviors due to the perception that violence is an inevitable part of their work that they should know how to cope with (54). This belief may compound the deleterious effects of exposure to violence and suggests a need for interventions that increase help-seeking behavior and coping strategies. Along these lines, our results highlight the efforts required to increase awareness of the negative impact of violence on MHCPs’ mental health, as well as programs to encourage their own mental health care and asking for support when needed (55, 56), especially among the younger MHCPs and those who experience physical violence during health emergencies.

### 4.4 Limitations and recommendations

One of the main limitations of this study is that we did not ask about the specific source of the violence (such as whether it was perpetuated by individuals outside the healthcare community, patients or patients´ relatives, co-workers, administrators or supervisors). Future studies analyzing this information, especially among the most vulnerable groups of healthcare workers (such as younger and early career professionals) would be useful for developing and implementing specific programs to prevent or mitigate occupational violence and protect the mental health care of this population during health emergencies such as COVID-19.

Other potential limitations of this study that could affect the generalizability of the sample include the use of an internet-based study, the recruitment of participants from an online network of MHCPs, and the fact that the response rate was 25.8%. Unfortunately, for the nearly 75% of GCPN members who chose not to participate in the study we do not have their demographic data or their reasons for not participating. Moreover, although we used an OV definition encompassing both physical and non-physical incidents, the prevalence of OV in our sample may be an underestimate, given people are reluctant to report experiences of violence, as has been reported in several studies of healthcare workers in general (31) and of psychiatry residents and practicing psychiatrists in particular (57). Moreover, data obtained for homicide rates were not available for all countries in the year of implementation of this study, although most cases (95.8%) are very close to the date of implementation (2018: 12.6%, 2019: 34.9%, and 2020: 48.3%).

Additionally, although the questions regarding the experience of each type (physical or psychological) and source (inside or outside the workplace) of OV clarified that these acts were related to their role as a health professional (see 2.2. variables and measures, line 145-147: “During the COVID-19 pandemic, have you been the target of physical or psychological violence or maltreatment, including being stigmatized or discriminated against because of your role as a health professional)?, there is a chance that the participants reported outside violence that was not indeed OV related.

Furthermore, although we attempted to control for differences between countries using national income level, homicide rates, COVID death rates, and the stringency of COVID-related controls, this did not control for more local variation (such as being in more violent neighborhoods with especially high COVID death rates).

### 4.5 Conclusions

In this international cross-sectional Internet-based study, completed by a large sample of MHCPs providing direct clinical services during the COVID-19 pandemic, approximately one in ten experienced OV. The most experienced type and source of OV was psychological violence inside the workplace. Risk factors for OV included being younger, working in emergency services, treating COVID-19 patients, and living in a lower to upper middle-income country. OV exposure was associated with significant emotional distress. Risk factors for emotional distress among those reporting OV included being younger and having experienced physical violence outside the workplace. These findings demonstrate the need for special efforts to prevent and manage OV, especially in the aforementioned high-risk groups of MHCPs, considering both the individual, proximal and distal contextual factors involved in the origin of OV toward them during health emergencies.

## Data Availability

The dataset presented in this article is not readily available for ethical/privacy reasons. Questions about access to the dataset should be directed to Dr. Cary Kogan: ckogan@uottawa.ca.
